# Travel and the emergence of high-level drug resistance in *Plasmodium falciparum* in southwest Uganda: results from a population-based study

**DOI:** 10.1186/s12936-017-1812-1

**Published:** 2017-04-17

**Authors:** Caroline A. Lynch, Richard Pearce, Hirva Pota, Connie Egwang, Thomas Egwang, Amit Bhasin, Jonathan Cox, Tarekegn A. Abeku, Cally Roper

**Affiliations:** 1 Faculty of Epidemiology and Population Health, London School of Hygiene and Tropical Medicine, London, UK; 2 Faculty of Infectious and Tropical Diseases, London School of Hygiene and Tropical Medicine, London, UK; 3 Medical Biotechnology Laboratories, Kampala, Uganda; 4 Malaria Consortium, London, UK

**Keywords:** Malaria, dhfr, Sulfadoxine–pyrimethamine (SP), Migration, Travel, Drug resistance

## Abstract

**Background:**

The I164L mutation on the *dhfr* gene confers high level resistance to sulfadoxine–pyrimethamine (SP) but it is rare in Africa except in a cluster of reports where prevalence >10% in highland areas of southwest Uganda and eastern Rwanda. The occurrence of the *dhfr* I164L mutation was investigated in community surveys in this area and examined the relationship to migration.

**Methods:**

A cross-sectional prevalence survey was undertaken in among villages within the catchment areas of two health facilities in a highland site (Kabale) and a highland fringe site (Rukungiri) in 2007. Sociodemographic details, including recent migration, were collected for each person included in the study. A total of 206 *Plasmodium falciparum* positive subjects were detected by rapid diagnostic test; 203 in Rukungiri and 3 in Kabale. Bloodspot samples were taken and were screened for *dhfr* I164L.

**Results:**

Sequence analysis confirmed the presence of the I164L mutations in twelve *P. falciparum* positive samples giving an estimated prevalence of 8.6% in Rukungiri. Of the three parasite positive samples in Kabale, none had I164L mutations. Among the twelve I164L positives three were male, ages ranged from 5 to 90 years of age. None of those with the I164L mutation had travelled in the 8 weeks prior to the survey, although three were from households from which at least one household member had travelled during that period. Haplotypes were determined in non-mixed infections and showed the *dhfr* I164L mutation occurs in both as a N51I + S108N + I164L haplotype (n = 2) and N51I + C59R + S108N + I164L haplotype (n = 5). Genotyping of flanking microsatellite markers showed that the I164L occurred independently on the triple mutant (N51I, C59R + S108N) and double mutant (N51I + S108N) background.

**Conclusions:**

There is sustained local transmission of parasites with the *dhfr* I164L mutation in Rukungiri and no evidence to indicate its occurrence is associated with recent travel to highly resistant neighbouring areas. The emergence of a regional cluster of I164L in SW Uganda and Rwanda indicates that transmission of I164L is facilitated by strong drug pressure in low transmission areas potentially catalysed in those areas by travel and the importation of parasites from relatively higher transmission settings.

## Background

The antifolate sulfadoxine–pyrimethamine (SP) is widely used for intermittent preventive treatment of malaria in pregnant women (IPTp) and was recently recommended for intermittent preventive treatment in children and infants (IPTi) [1]. In addition, another antifolate, co-trimoxazole (trimethoprim–sulfamethoxazole), is recommended by the World Health Organization (WHO) for treatment of childhood febrile diseases and for prophylaxis against opportunistic infections in HIV-infected patients in Africa. Commonly occurring mutations at codons 51, 59, 108 and 164 of the *dhfr* gene of malaria parasite *Plasmodium falciparum* are associated with resistance to SP, and cumulatively they confer increasingly high levels of resistance. The triple mutant haplotype N51I, C59R and S108 N has been long established in Africa, but the combination of all 4 mutations remains rare despite the fact that it confers a higher level of resistance and became established in Southeast Asia as early as 1997 [2].

A review of published literature in 2012, identified 183 surveys in 114 unique geographical localities in Africa, where *P. falciparum* isolates were tested for mutations at codon 164 of *dhfr*. Of 19,597 isolates tested, just 130 (0.7%) were positive for I164L [3]. The mutation occurs at low level in multiple locations; 3% prevalence in western Kenya [4, 5]; 4% in Blantyre, Malawi [6]; 0.6% in the Central African Republic [7]; 1.1% in Comoros [8]; 1.0% in southern Madagascar [8] and 2% in Angola [9]. So far, it only occurs in significant numbers in Uganda [10, 11] and Rwanda.

**Fig. 1:**
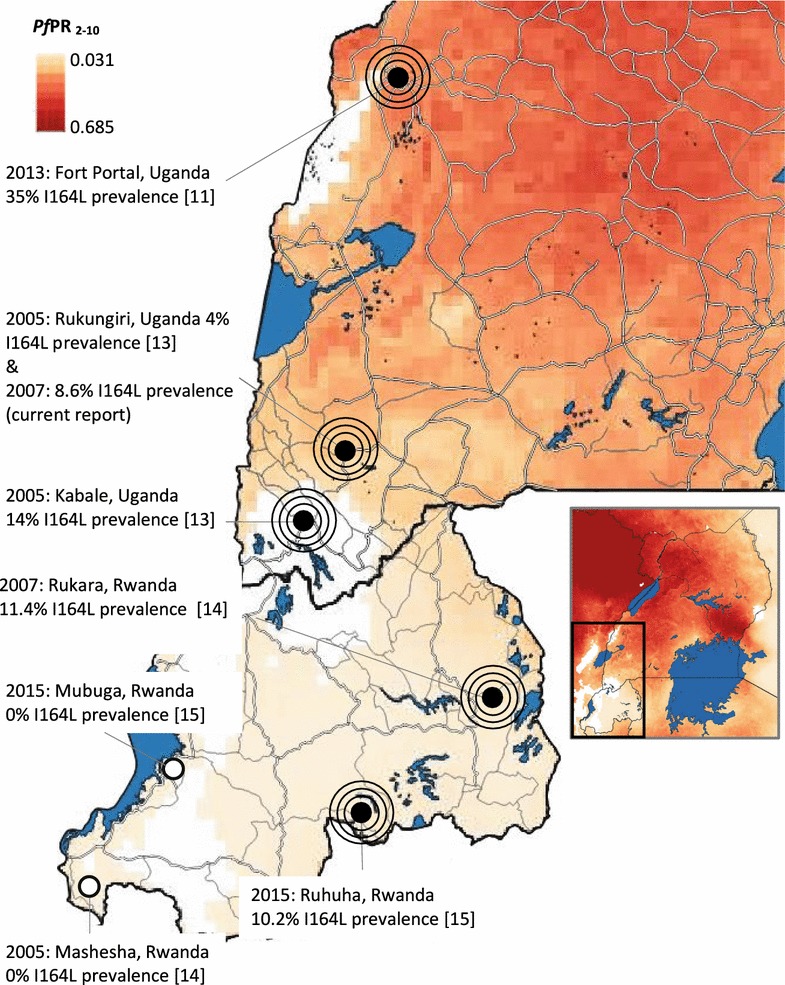
Percentage prevalence of I164L mutation detected in Uganda 2003–2015 shown (*black* and *white circles*) with place name, year of sampling, study reference, roads (*white lines*) and estimated transmission risk [28]

**Fig. 2:**
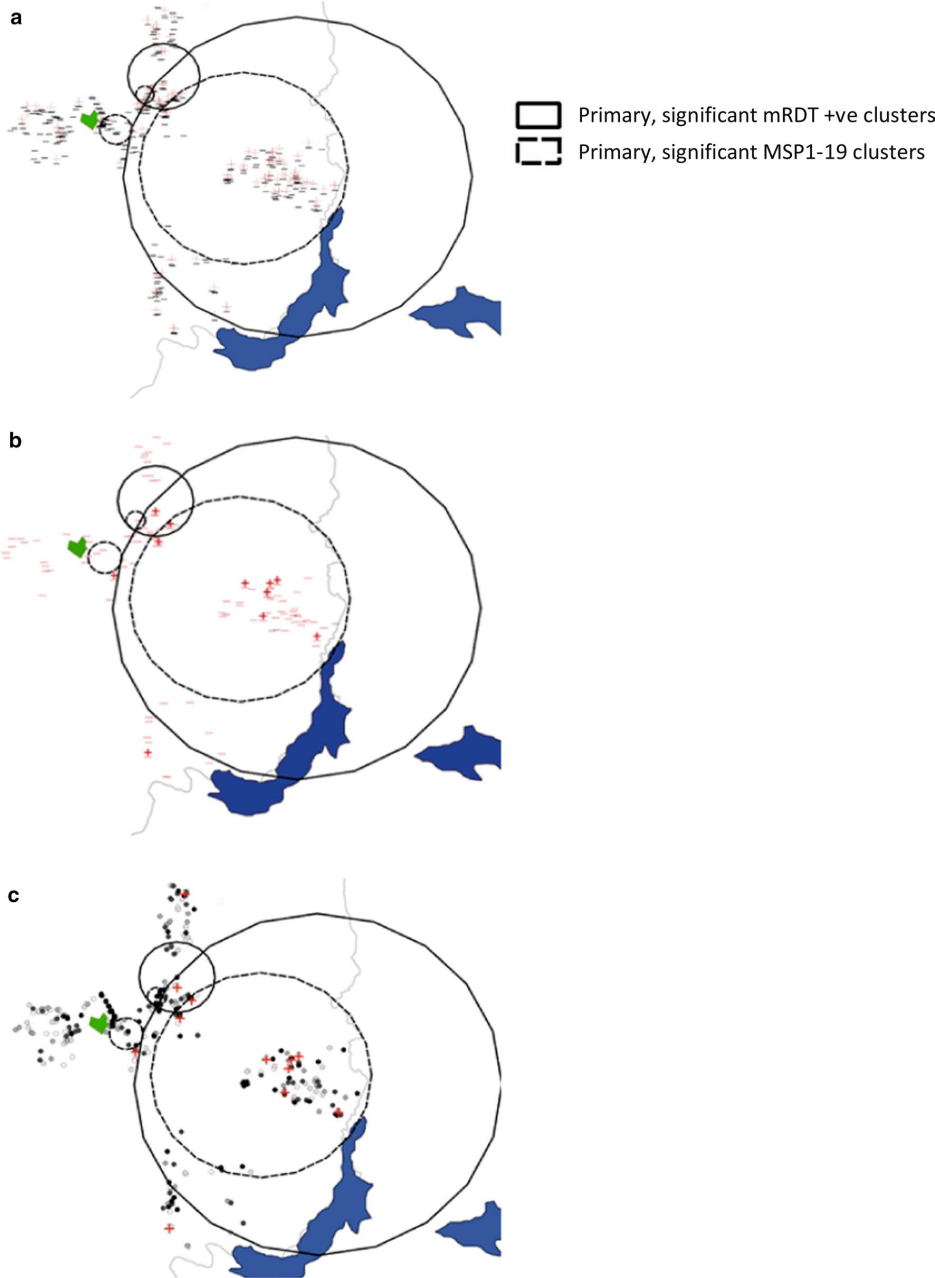
**a**–**c** Malaria rapid diagnostic test results, MSP1_19_ seroprevalence and I164L prevalence in Rukungiri district, Uganda

Early surveys for the I164L mutant in Uganda in 2002–2004, found just a single I164L mutation among 480 isolates from six sites across Uganda [12]. The I164L mutant was 1 among 80 pre-treatment samples (mutation prevalence, 1.25%) from a clinic in Kanungu in southwest Uganda. By 2005, prevalence of I164L in southwest Uganda was increasing. Lynch et al. looked at I164L among malaria patients attending health facilities in highland and fringe highland areas of southwest Uganda in 2005 [13]. The I164L mutation was found at 14% prevalence in Kabale (highland) and 4.2% prevalence in Rukungiri (fringe highland) [13]. In the same year malaria patients attending a clinic in Rukara in neighbouring Rwanda were found to have 11.4% prevalence of I164L [14]. Rukara is just 120 km from Kabale and 200 km from Rukungiri suggesting a common source of I164L. Patients in Mashesha, western Rwanda which is 400 km from Kabale, Uganda were screened in the same study, and no I164L was present [14] (see Fig. 1) supporting the view that 164L had a restricted focal distribution. More recent surveys in clinics 2015 in Mubuga in western Rwanda (0% prevalence) and Ruhuha in Eastern Rwanda (10.2% prevalence) confirm that this is still the case [15] (see Fig. 2). This cluster of reports of elevated prevalence of I164L in clinical isolates from SW Uganda [13] and bordering Eastern Rwanda [14] supports the view that focal transmission of highly resistant malaria occurs in the region. However, in each of these studies the reported prevalence of I164L was among patients attending clinics with confirmed cases of symptomatic malaria and these may have been home treated with SP.

To know the extent of the mutation in the parasite reservoir in SW Uganda, cross sectional community surveys were carried out to detect and screen asymptomatic infections for I164L and determine the baseline prevalence of this mutation. Surveys had previously been conducted in 2007 among communities in the catchment area of the health facilities where malaria patients were originally screened in 2005 [13]. The prevalence of the I164L mutation was measured among asymptomatic infections, and looked at the number of *dhfr* resistance lineages from which these were derived. The travel histories of parasite positive cases with I164L and without I164L mutation were recorded to examine the extent to which migration or travel outside the study areas may explain the occurrence of infections with I164L.

## Methods

### Study sites

A cross-sectional prevalence survey was undertaken in villages within the catchment areas of two health facilities in Kabale and Rukungiri districts in 2007. Four villages in each district were randomly chosen and surveyed in their entirety. The primary sampling unit for the study was the household. All households in the selected village who had members present, and/or consented to the study were surveyed. Study sites are described elsewhere [16]. Kabale is a highland area (mean altitude 2200 m) where only three parasite positive samples were detected out of 911 sampled at the time of the study. Rukungiri is a fringe highland (mean altitude 1500 m) area in Uganda where the mean parasite positivity rate based on cross-sectional community surveys for *P. falciparum* was 12.1% (95% CI 10.0–14.2) at the time of the study [16].

### Collection of travel data

A structured household questionnaire was used to capture information on household residents and visitors’ sociodemographic, travel and malaria control and prevention in the household. Specific to mobility, participants were asked details of their travel outside their sub-county of residence during the 8 weeks prior to the survey. As well as information on whether the participant travelled, they were also asked their dates of departure and return to estimate duration of exposure, their destination sub-county and district, their reason for travel and whether they used an insecticide-treated net when travelling.

### Sample collection and laboratory analysis

Bloodspots for samples were taken on Whatman^®^ 3MM filter paper, dried and stored with silica gel. Out of 2125 samples collected in both sites, 206 subjects found Paracheck^®^ positive. Samples were analysed for the I164L mutation on *dhfr* using methods described in Lynch et al. [13]. DNA was prepared from bloodspots using a Chelex extraction method and PCR amplification of *dhfr* carried out as previously described [17]. Amplified PCR products were screened for *dhfr* sequence variants at five loci where single nucleotide polymorphisms (SNPs) are known; *dhfr* codons 50, 51, 59, 108, 164. PCR products were spotted in a 12 by 8-grid and cross-linked onto nylon membranes and probed for sequence polymorphisms by hybridization to specific oligonucleotide probes as described previously [17]. Probed blots were visualized using ECF substrate and detection using a phosphoimager (GE Healthcare, Buckinghamshire, UK). Output was recorded through viewing of digitally captured images of chemifluorescent signal.

The presence, absence, and relative abundance of hybridization signal was recorded for every probe at each locus. A sample was considered to have a single haplotype when only one sequence variant was found at each locus. Blood samples were categorized as having a single, a majority or mixed form of sequence at every SNP locus. Majority and mixed genotype infections were differentiated according to the relative intensity of signal.

Polymorphic microsatellite repeats were studied in the flanking region of *dhfr* on chromosome 4. Microsatellite markers located 0.3, 4.4 and 5.3 kb upstream from codon 108 were analysed, amplifying each locus by PCR then measuring fragment size on an ABI 3730 sequencer and using the software Genemapper (Applied Biosystems, Warrington, Cheshire, UK). A semi-nested PCR design was used and the primer sequences and PCR reaction conditions are described elsewhere [18].

### Ethics, consent and permissions

Ethical clearance was obtained from Ugandan National Council for Science and Technology (HS-35) and London School of Hygiene & Tropical Medicine (3053). In addition, the blood sampling was demonstrated to Local Chairmen (LC) and executive committees of each village surveyed, and approval for the survey sought. Informed consent was obtained from the head of household for all human adult participants as well as any children under 18. Parasite positive individuals were referred to the local health facility to receive free treatment.

## Results

### Prevalence of molecular markers for drug resistance at community level

Only three parasite positive samples were found in Kabale and none of these had the I164L mutation. The prevalence of point mutations in the Rukungiri samples is shown in Table 1.

**Table 1:** Prevalence of wild-type and mutant codons in the *dhfr* genes of *Plasmodium falciparum* among subjects sampled during cross-sectional community survey, 2007 [16]

Gene, codon	Amino acid	Prevalence (%) Rukungiri
Wild-type		
51	N	0/139 (0)
59	C	5/139 (4)
108	S	0/139 (0)
164	I	131/139 (94)
Mutant		
51	I	139/139 (100)
59	R	134/139 (96)
108	N	139/139 (100)
164	L	12/139 (8.6)

Sequence analysis confirmed the presence of the I164L mutations in 12 of the 139 *P. falciparum* positive samples from Rukungiri from which DNA was successfully amplified, giving an estimated I164L prevalence of 8.6% (n = 12/139).

Haplotypes could be confidently determined in isolates which did not have mixed signal in any of the codons. The haplotype frequencies among this subset of samples are shown in Table 2. There was a predominance of the triple mutant haplotype IRNI (N51I, C59R + S108N) a small number of double mutant haplotypes (S108N + N51I), and zero sensitive/non-mutant haplotypes. Seven haplotypes containing the I164L could be confidently determined. In two cases the I164L was on an ICNL haplotype (S108N + N51I + I164L) and in five cases on an IRNL (S108N + C59R + N51I + I164L).

**Table 2:** Point mutation haplotype frequencies in the *dhfr* genes [proportion (%) of positive subjects], 2007

Haplotype	Rukungiri
NCSI	0/128 (0)
**IRN**I	120/128 (94.5)
**I**C**N**I	1/128 (1.6)
**I**C**NL**	2/128 (2.3)
**IRNL**	5/128 (1.6)

Polymorphism at the three flanking microsatellite loci is shown in Table 3. *Dhfr* mutations from the 2007 community survey are shown with their associated microsatellite flanking haplotypes and they are compared *dhfr* mutant alleles in clinical isolates from 2005 (13). Allele sizes at the microsatellite loci show the triple mutant **IRN**I haplotype is associated with microsatellite 0.3/4.4/5.3 kb flanking haplotype 108/176/203. This was true in both 2005 and 2007 and is confirmation that this mutant allele derives from the common triple mutant lineage found throughout Africa. The IRNL haplotype was associated with the same microsatellite flanking haplotype 108/176/203 indicating that the I164L was additionally acquired onto the triple mutant lineage. Interestingly the double mutant **I**C**N**I has several independent, local origins each different from the triple mutant.

**Table 3:** Microsatellite haplotypes associated with the *dhfr* mutant alleles found in 2005 and 2007

Haplotype	0.3 kb	4.4 kb	5.3 kb	2005	2007
1	87	172	205		2 **I**C**NL** ^a^
2	87	176	193		1 **IRN**I
3	87	176	203	1 **I**C**N**I	
4	87	176	210	1 **I**C**N**I	
5	87	178	193	1 **I**C**N**I, 1 **I**C**NL** ^a^	1 **I**C**N**I
6	108	176	193	1 **IRN**I	2 **IRN**I
7	108	176	199		1 **IRN**I
8	108	176	201		1 **IRN**I
9	108	176	203	57 **IRN**I + 6 **IRNL** ^a^	49 **IRN**I + 4 **IRNL** ^a^
10	108				1 **IRNL** ^a^
11	108	176	209		1 **IRN**I
12	108	176			4 **IRN**I
13	108		203		1 **IRN**I
14	110	176	201		1 **IRN**I
15	110	176	203		3 **IRN**I
16	110	176			1 **IRN**I
17	112	176	203	2 **IRN**I	

Fragment sizes are shown in base pairs and are standardised across the 2 years by running fragments on the same machine with identical size standard

^a^I164L mutations

In 2007, the ICNL mutant haplotype was found in association with microsatellite haplotype 87/172/205 while in 2005 it was linked to haplotype 87/178/193. A number of microsatellite haplotypes are commonly associated with the **I**C**N**I double mutant in East Africa and the I164L mutation has clearly been acquired independently on at least two of these lineages.

Travel data was available for all 12 subjects with the I164L mutations; none had travelled in the 8 weeks prior to the survey. Two of the four villages surveyed were where most of the I164L mutations were found with five people from one village living within 600 m of each other (which was identified as a cluster for *P. falciparum* positive subjects). In the second village, three subjects lived within 550 m distance of each other while another I164L positive person lived approximately 1.8 km away (Fig. 2a–c). The two remaining villages surveyed each had one person with positive for I164L. Four of those surveyed were from households in which there were other parasite positive individuals (Table 4). Nine were female, three were male and ages ranged from 5 to 90 year of age.

**Table 4:** Demographic and travel history for twelve subjects for which an I164L *Plasmodium falciparum* mutant was recovered

Altitude	Age	Sex	Travel <8 weeks	HH member travel last 8 weeks	Total HH members +ve out of those sampled
1551–1600	5	M	N	N	1/4
1551–1600	10	F	N	N	1/5
1500–1550	9	F	N	Y	3/7
1500–1550	10	F	N	Y	1/5
1450–1499	5	F	N	N	1/4
1450–1499	6	M	N	Y	1/3
1450–1499	13	F	N	N	1/7
1400–1450	5	F	N	N	4/4
1400–1450	10	F	N	N	1/1
1400–1450	29	F	N	N	3/7
1400–1450	90	F	N	N	2/5
1350–1399	7	M	N	N	1/7

## Discussion

The I164L mutation on the *dhfr* gene is known to confer high level resistance to SP but it is rare in African *P. falciparum* except in southwest Uganda and bordering areas of eastern Rwanda. Previously all the I164L mutation prevalence reports from this geographical area had been measured in patient isolates collected at health facilities. One possible explanation for the higher rate of I164L among symptomatic infections is that there was prior selection by home or community treatment before attendance at the clinic. In Uganda, at the time of the study, there was a policy of home treatment with chloroquine-SP (CQ + SP) branded as ‘Homapak’ which could have pre-selected resistance mutations prior to clinic attendance. Although home treatment via ‘homapak’ was not policy in Rwanda, CQ + SP was national first-line treatment between 2001 and 2005 and widely available through both public and private sector facilities [19].

In this current study, community surveys were carried out to identify the reservoir of *P. falciparum* infection and test these parasites for *dhfr* I164L. It was confirmed that the parasite reservoir around Rukungiri clinic the I164L mutation occurs at a prevalence rate of 8.6%. This is in fact higher than was recorded among patients attending Rukungiri clinic in 2005 among whom the prevalence of I164L was 4.2%. Based on these findings, conclusions are that the I164L mutations in Rukungiri malaria infection reservoir has been sustained or has slightly increased over the 2 years between sampling in 2005 and 2007.

A higher prevalence of I164L was found among patients in Kabale in 2005, but the community survey of 2007 managed to identify only 3 infections because transmission intensity is very low. There are previous reports of an association between malaria infection in Kabale residents with recent travel to higher transmission areas particularly to the region north of Kabale [20, 21] where the tea estates around Fort Portal in western Uganda draw a significant seasonal labour workforce (see Fig. 2). It is possible that the I164L mutation was imported to Kabale by seasonal migrants returning from that region. A prevalence of 36% *dhfr* I164L was recorded in 2013 among pregnant women (n = 55) receiving SP-IPTp [11] This indicates that I164L is abundant in the parasite population there, although clearly the prevalence estimates will be elevated among populations receiving SP prophylaxis.

Microsatellite analysis from 2007 samples in the study sites indicate similar results to the 2005 analysis (12) in that the I164L mutation has occurred on the triple mutant ‘IRNI’ (N51I + C59R + S108N) background as well as on at least 2 *dhfr* double mutant lineages ICNI (N51I + S108N). Strong drug pressure is the most likely explanation for the high occurrence of I164L, and its repeated de novo emergence in this area. A further possibility however is the emergence of adaptations in other genes in the same pathway, which can compensate for deleterious effect of the 164L mutation in these populations. Increased copy number of the GTP cyclohydrolase (*gch1*) gene is strongly associated with the *dhfr* 164L mutation in southeast Asia [22]. Duplications in the promotor of gch1 are found in East Africa and reported to be particularly common in Malawi [23] although not found in association with 164L. Further exploration of copy number variation in genes of the folate biosynthesis pathway in parasites of SW Uganda and Eastern Rwanda is warranted. Additional investigations are warranted, in particular examination of contemporary samples collected from this particular geographical area.

In general, stronger drug selection is expected in areas of lower transmission because in less malaria-exposed populations a higher proportion of infections are symptomatic and therefore more likely to be treated with anti-malarial drugs. Transmission intensity is very low in the Kabale highlands, but in the highland fringe areas of Fort Portal, Kanungu, Rukungiri, Rukara and Ruhuha transmission is considered to be meso- to hyper-endemic with localized variation across the region [24] (see Fig. 1). Local transmission occurs in these areas, this is reflected in the age distribution of infection and the incidence profile of malaria disease in these communities.

The strength of selection for SP resistance will have changed since the first report of I164L in 2003. In Uganda the first-line recommended treatment was changed in 2006 from SP + CQ to artemether + lumefantrine [25], although use of SP continued throughout the transition to artemisinin-based combination therapy (ACT), and SP continues to be used in IPTp to the present day. In Rwanda, implementation of ACT as first-line treatment also started in 2006 during which a complete changeover was made from SP + CQ to ACT alone [26]. The prevalence of I164L mutations appears to be increasing nevertheless which suggests the selection of I164L is continuing and perhaps it may be promoted by the high incidence of symptomatic infections in highland populations.

It is clear from this and other molecular surveillance studies carried out since 2003 that a highly SP resistant sub-population of *P. falciparum* exists and is maintained in the highland fringe areas extending from Rwenzori mountains in Uganda down into eastern Rwanda. The northern limits of its distribution are yet to be determined but it is absent in western Rwanda and this pattern of distribution suggests that a particular combination of factors such as selection and migration promote and maintain the I164L in this area. With increasing investment in road infrastructure throughout sub-Saharan Africa [27], and in the Great Lakes region in particular, the risk of spread of highly resistant mutations is higher than ever before. It will be important to monitor the spread of highly resistant I164L mutants in Uganda in particular. Previous work has shown that gene flow has played a central role in the establishment of resistance to CQ and SP at sites across Africa, so the spread of mutants has the potential of becoming a wider problem. Further analysis of parasites from the western Uganda, and from Eastern areas of DR Congo bordering both Rwanda and Uganda would determine the geographical extent of the I164L cluster.

## Conclusions

Findings in 2005 and in this currently reported 2007 work demonstrate the prevalence of I164L in demographically different populations from those normally tested for I164L. In our 2005 study, patients presenting were men between 18 and 30 years who had travelled <6 weeks previously, while in the community survey presented here those with I164L mutations were aged between 5 and 90 years and were both male and female and had not travelled recently (Table 4). In further investigations aiming to define the limits of the sub-region affected by the highly SP resistant parasites it will be necessary to survey at population level, with particular attention to seasonal or circular migrants. Not just on the more commonly undertaken studies screening pregnant women on IPTp or children <5 years recruited to anti-malarial efficacy studies.

The sustained presence of, and even increase in, I164L mutations in the sub-region even after the introduction of ACT, strongly indicates the need for a targeted approach to the control of malaria in pregnancy. Given the likely presence of I164L, screening and treating confirmed malaria cases in pregnancy would be more appropriate than SP-IPTp in the affected region.

The emergence of a regional cluster of I164L in SW Uganda and Rwanda indicates that transmission of I164L is facilitated by strong drug pressure in low transmission areas and potentially catalyzed in some areas by travel between relatively higher to lower transmission settings. Attention to treatment policy on the fringes of malaria distribution is particularly important in the context of malaria elimination zones on the African continent.

## Abbreviations

Dhfr: dihydrofolate reductase
Kb: kilobytes
SP: sulfadoxine–pyrimethamine
CQ: chloroquine
ACT: artemisinin-based combination therapy
